# Impact of the North American Free Trade Agreement on high-fructose corn syrup supply in Canada: a natural experiment using synthetic control methods

**DOI:** 10.1503/cmaj.161152

**Published:** 2017-07-04

**Authors:** Pepita Barlow, Martin McKee, Sanjay Basu, David Stuckler

**Affiliations:** Department of Sociology (Barlow), University of Oxford, Oxford, UK; Department of Public Health and Policy (McKee), London School of Hygiene and Tropical Medicine, London, UK; Stanford Prevention Research Center (Basu), Stanford University, Palo Alto, Calif; Department of Policy Analysis and Public Management (Stuckler), Bocconi University, Milan, Italy.

## Abstract

**BACKGROUND:**

Critics of free trade agreements have argued that they threaten public health, as they eliminate barriers to trade in potentially harmful products, such as sugar. Here we analyze the North American Free Trade Agreement (NAFTA), testing the hypothesis that lowering tariffs on food and beverage syrups that contain high-fructose corn syrup (HFCS) increased its use in foods consumed in Canada.

**METHODS:**

We used supply data from the Food and Agriculture Organization of the United Nations to assess changes in supply of caloric sweeteners including HFCS after NAFTA. We estimate the impact of NAFTA on supply of HFCS in Canada using an innovative, quasi-experimental methodology — synthetic control methods — that creates a control group with which to compare Canada’s outcomes. Additional robustness tests were performed for sample, control groups and model specification.

**RESULTS:**

Tariff reductions in NAFTA coincided with a 41.6 (95% confidence interval 25.1 to 58.2) kilocalorie per capita daily increase in the supply of caloric sweeteners including HFCS. This change was not observed in the control groups, including Australia and the United Kingdom, as well as a composite control of 16 countries. Results were robust to placebo tests and additional sensitivity analyses.

**INTERPRETATION:**

NAFTA was strongly associated with a marked rise in HFCS supply and likely consumption in Canada. Our study provides evidence that even a seemingly modest change to product tariffs in free trade agreements can substantially alter population-wide dietary behaviour and exposure to risk factors.

Trade between Canada and the United States has been transformed over the past 3 decades.^1^–^4^ Amid considerable controversy, the Canada–United States Free Trade Agreement (CUSFTA) came into force in 1989, followed by the North American Free Trade Agreement (NAFTA) in 1994 (Box 1).^1^ Although CUSFTA removed most tariffs on goods and services, some restrictions remained, including those on high-fructose corn syrup (HFCS).^2^,^5^ However, the remaining tariffs were removed progressively once NAFTA was signed.^6^–^9^

Box 1: **Key terms and acronyms**

**Free trade agreement (FTA):** A major policy tool for reducing tariffs (a trade tax) and nontariff barriers to trade and investment.

**The North American Free Trade Agreement (NAFTA):** A trade deal between Canada, Mexico and the United States that came into force on Jan. 1, 1994. NAFTA superseded an earlier agreement between Canada and the US in 1989, the Canada–United States Free Trade Agreement (CUSFTA).

**High-fructose corn syrup (HFCS)**: A form of sugar that is linked to the development of noncommunicable diseases and risk factors, including dyslipidemia, cardiovascular disease, metabolic syndrome, obesity and diabetes.^8^,^9^,^19^,^51^–^54^

Public health commentators have long expressed concern that NAFTA, as with other free trade agreements (FTAs), could pose a threat to health.^10^–^12^ Yet, empirical research on the consequences of NAFTA for the health of Canadians is limited, with most previous studies focused on its economic and political impacts.^4^,^13^ One prominent concern centres on lower prices created by trade liberalization, including lower tariffs, which lead to increased imports of energy-dense products that lack nutritional benefits: so-called “empty calories.”^14^,^15^ Lower prices encourage manufacturers to use these products in cheap processed food, with consequences for obesity and the health effects that flow from it.^11^

Although these concerns have been voiced repeatedly, concrete evidence has been limited thus far.^16^,^17^ Most previous studies of FTAs and diets are single-country case studies and preclude conclusions about causality.^18^,^19^ Difficulties in estimating the impact of FTAs are compounded by their complexity, as FTAs include changes to both tariff and nontariff barriers applicable to both trade and investment.^20^ The existing literature has been unable to untangle this complexity.^19^ Many studies are sensitive to sample selection, and very little attention has been paid to specific potentially harmful foods, such as HFCS, which is linked to dyslipidemia, cardiovascular disease and metabolic syndrome (Box 1).^8^,^9^,^19^,^51^–^54^ Here we take advantage of an exceptional natural experiment in which tariffs on HFCS were withdrawn, within an existing system of free trade in goods, to study the effect of withdrawal of tariffs on HFCS products on consumption of the same in Canada.

## Methods

We use an innovative methodology of time-series analysis with a “synthetic control” that estimates the supply in Canada against a control group, including specific countries and a weighted combination of comparison control nations. This better simulates experimental conditions to assess the null hypothesis of no effect, or what would have happened to the supply of food and beverage syrups that include HFCS in the absence of a change to import tariffs.

### Study setting

NAFTA included a schedule that set out subtle but potentially important changes to tariffs on Canadian imports of caloric HFCS syrups used in food and beverage production between 1994 and 1998, as shown in Appendix 1 (Appendix 1, available at www.cmaj.ca/lookup/suppl/doi:10.1503/cmaj.161152/-/DC1). A longstanding dispute over subsidies to US cane and beet sugar farmers had prevented tariffs on all food and beverage syrups from being removed in CUSFTA.^2^,^5^ The concerns were mainly with respect to cane and beet sugars, but syrups from cane and beet sugars and HFCS were combined in 1 tariff category. However, NAFTA’s tariff schedule separated them into 2 categories, so tariffs on food and beverage syrups containing HFCS were gradually removed between 1994 and 1998, but remained in place for cane and beet syrups.^21^–^23^

### Data sources and measures

To evaluate the effect of the tariff reductions arising from NAFTA, we use annual food supply data from the Food and Agriculture Organization of the United Nations Statistics Office (FAOSTAT).^24^ FAOSTAT data comprise the only available data source for estimating changes in food consumption on a comparative, cross-national basis during the study period. The FAOSTAT database details total food supply for human consumption in kilocalories (kcal) per capita per day. Annual supply figures are the sum of imports and domestic production, less exports, estimated wastage and stocks from the previous period. Table 1 summarizes the definitions of variables and the data sources used in our analysis. Governments report food supply data to the United Nations based on harmonized classification and measurement guidelines. Supply of HFCS is captured in FAOSTAT’s measure of total caloric sweeteners, which also captures additional syrups with unchanged tariffs: pure fructose and maltose, maple sugar and syrup, glucose, dextrose, lactose and molasses, although the category is not disaggregated further.^28^

**Table 1:** Summary of variable definitions and data sources

Variable	Measure	Source
Supply of caloric sweeteners including HFCS, cane and beet sugars, total sugars, cereals, fruits, meats, vegetables, vegetable oils, animal fats and all food	Kilocalories per capita	Food and Agriculture Organization of the United Nations Statistics Office
US exports of HFCS beverage and other sugar syrups to Canada	Net exports (exports to Canada less imports and re-exports to the US) in kilograms per capita*	United States Department of Agriculture
Income	GDP per capita measured in constant 2005 US dollars and adjusted for differences in purchasing power	World Bank World Development Indicators (2015 edition)
Inflation	Annual percent growth rate of the GDP implicit deflator (the ratio of GDP in current local currency to GDP in constant local currency)	World Bank World Development Indicators (2015 edition)
US investments in Canadian corn syrup industry	Indicators of the establishment of Canadian branches or acquisition of Canadian companies by major US corn syrup producers	Investment data from the Investment Review Division, Government of Canada; list of major US corn syrup producers from US Corn Refiners Association

Note: GDP = gross domestic product, HFCS = high-fructose corn syrup.

* US exports are reported as totals and were converted to per capita figures using population estimates from the World Bank World Development Indicators, 2015 edition.^24^–^27^

Our models adjust for urbanization and for national income (gross domestic product [GDP] per capita corrected for differences in prices and in purchasing power) using data from the World Bank World Development Indicators 2015 edition.^25^ Our models also adjust for differences in dietary preferences using FAOSTAT measures of vegetable, vegetable oil, fruit, meat, cereal, animal fat and total food consumption. We examine changes in US exports of HFCS using data from the United States Department of Agriculture Global Agricultural Trade System database.^26^

### Fixed effects and synthetic control models

To estimate the effect of NAFTA, we are interested in comparing levels of supply in Canada with its control. We first estimate cross-national fixed-effects regression models with country-specific intercepts; i.e., dummy variables for each country that capture unobserved, time-invariant country characteristics that could affect caloric sweetener supply. We compare Canada with 2 other high-income countries that approximate this control, as they had parallel trends in supply before NAFTA: Australia and the United Kingdom (Appendix 2, available at www.cmaj.ca/lookup/suppl/doi:10.1503/cmaj.161152/-/DC1).

Because fixed-effects regression models can yield biased effect estimates, we then estimate the control for Canada using the “synthetic control” method.^29^,^30^ This approach overcomes certain limitations associated with the widely used Difference-in-Differences approach.^31^ It estimates supply in Canada’s control from a weighted combination of comparison countries that were similar (as identified using variables that predict supply), but were not exposed to US FTAs. Weights are assigned according to each country’s similarity with Canada pre-NAFTA and each variable’s predictive power (see Appendix 3 [available at www.cmaj.ca/lookup/suppl/doi:10.1503/cmaj.161152/-/DC1] for a full description of this method).^32^,^33^ Following previous studies of dietary change and consumption, the predictors are GDP per capita and inflation, as well as vegetable, vegetable oil, fruit, meat, cereal, animal fat and total food consumption.^34^–^36^ We also include 1-year lagged values of the outcome variable to capture unobserved factors that affect supply.^37^

The pool of comparison countries is restricted to countries that enable us to distinguish the effect of NAFTA from other important macroeconomic and trade policy changes. Similar to Canada, the 16 comparison countries were members of the Organisation for Economic Co-operation and Development (OECD), and made similar global trade policy commitments as members of the World Trade Organization (WTO), and the General Agreement on Trade and Tariffs (the WTO’s precursor). However, unlike Canada, the “control group” countries did not enter a US FTA during the study period: Australia, Austria, Denmark, Finland, France, Germany, Greece, Italy, Japan, Netherlands, New Zealand, Portugal, Spain, Sweden, Switzerland and the UK. Appendix 4 (available at www.cmaj.ca/lookup/suppl/doi:10.1503/cmaj.161152/-/DC1) summarizes the countries used as comparison units and their weights from this analysis. The control for Canada is a weighted combination of all 16 countries in the donor pool. Four-fifths of Canada’s control is constructed using predictor observations in Spain, Germany and New Zealand, and the remaining fifth is based on the remaining 13 countries (Appendix 5, available at www.cmaj.ca/lookup/suppl/doi:10.1503/cmaj.161152/-/DC1). Our sample covers the years 1985–2000. Additional analyses confirmed that our results are not qualitatively affected by our choice of study period (Appendix 6, available at www.cmaj.ca/lookup/suppl/doi:10.1503/cmaj.161152/-/DC1). Analyses were done in STATA version 13.1.

### Methods for checking robustness

We carried out additional analyses to test whether our findings are sensitive to our sample specification and differences in trade instruments between North America and Europe. This included an “in-space” placebo used to estimate *p* values by comparing the effect estimated for Canada with a placebo effect obtained by iteratively reassigning NAFTA to countries that did not actually implement it, and then calculating placebo effects for each country.

### Ethics approval

No ethics approval was required for the study, as we used publicly available, pre-anonymized data.

## Results

**Figure 1:**
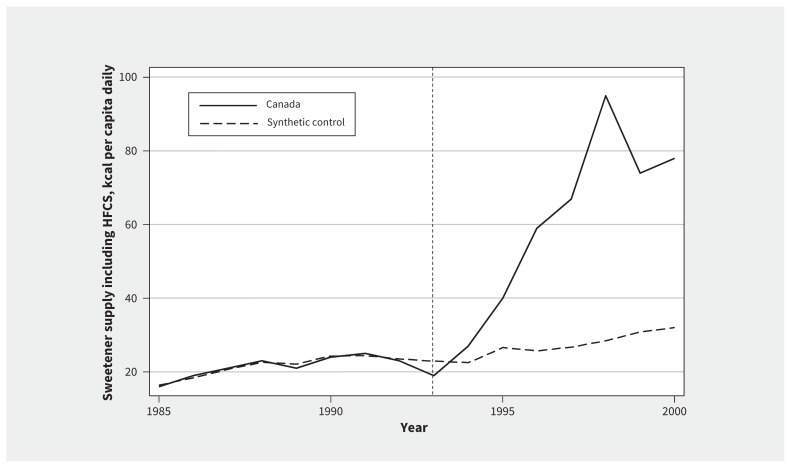
Trends in the supply of sweetener syrups including high-fructose corn syrup (HFCS) in Canada and the synthetic control, 1985–2000. Note: Vertical line shows the implementation of the North American Free Trade Agreement on Jan. 1, 1994.

Figure 1 shows that in Canada, the daily per capita supply of caloric sweeteners including HFCS rose from 21.2 kcal (95% confidence interval [CI] 10.26 to 32.19) in the pre-NAFTA period to 62.9 kcal (95% CI 50.43 to 75.29) post-NAFTA. This represents a 41.6 kcal (95% CI 25.06 to 58.21) increase after tariffs were removed on food and beverage syrups containing HFCS. The rise in caloric sweeteners persisted for each year that tariffs were reduced, stopping after the final reduction in 1998. Cross-national fixed-effects models also identify an increase in supply of caloric sweeteners after Canada acceded to NAFTA, compared with Australia and the UK (NAFTA estimate: 34.39 kcal, 95% CI 30.02 to 38.76; see Appendix 7, available at www.cmaj.ca/lookup/suppl/doi:10.1503/cmaj.161152/-/DC1).

The rise in supply of caloric sweeteners including HFCS coincided with an interruption in the long-term decline in total sugar and sweetener supply in Canada. Before NAFTA, total sugar supply was declining, mirroring cane and beet sugars. From 1994, trends in total sugars and cane and beet sugars diverged (Appendix 8, available at www.cmaj.ca/lookup/suppl/doi:10.1503/cmaj.161152/-/DC1). Supply of cane and beet sugars in Canada was on average 20.6 kcal/capita daily (95% CI 5.63 to −46.81) lower than before NAFTA, but supply of all sugars and sweeteners was, on average, 21.05 kcal/capita daily (95% CI 0.95 to 41.15) higher. Caloric sweeteners including HFCS accounted for an increasing proportion of total sugar and sweeteners, rising from 4.8% (95% CI 2.44 to 7.13) before NAFTA to 13.5% (95% CI 10.80 to 16.11) after NAFTA.

Appendix 9 (available at www.cmaj.ca/lookup/suppl/doi:10.1503/cmaj.161152/-/DC1) also shows that net Canadian imports of beverage syrups doubled in the period after NAFTA (1994–2000), rising from 7132.4 metric tons in 1993 to 16 062.0 tons in 2000 and claiming a growing share of total beverage syrup imports.

### Synthetic control results

Table 2 shows that the characteristics of Canada and the synthetic control before NAFTA were very similar in terms of predictors of caloric sweetener supply.

**Table 2:** Sweetener supply predictor means before NAFTA*

Predictor	Canada	Synthetic control
GDP per capita	27 216.25	27 186.20
Inflation	3.55	4.55
Supply, t-1	21.25	21.21
Cereals	657.75	770.68
Fruits	115.25	111.79
Meats	358.38	342.92
Vegetables	81.50	74.52
Vegetable oils	417.25	397.22
Animal fats	238.25	206.41
Total kcals	3060.38	3163.77

Note: GDP = gross domestic product, NAFTA = North American Free Trade Agreement, t-1 = lagged values in the previous year.

* GDP and urbanization data from the World Bank World Development Indicators (2016 edition). Supply data from FAOSTAT.

Figure 1 shows that the trend in supply of sweetener syrups was very similar in Canada and its control before NAFTA but, after NAFTA accession, the 2 diverge: supply accelerates in Canada, but in the control, estimated supply remains similar to that pre-NAFTA. On average, supply of caloric sweeteners was 43.7 kcal/capita daily higher in Canada compared with the control in the post-NAFTA period.

### Robustness checks

**Figure 2:**
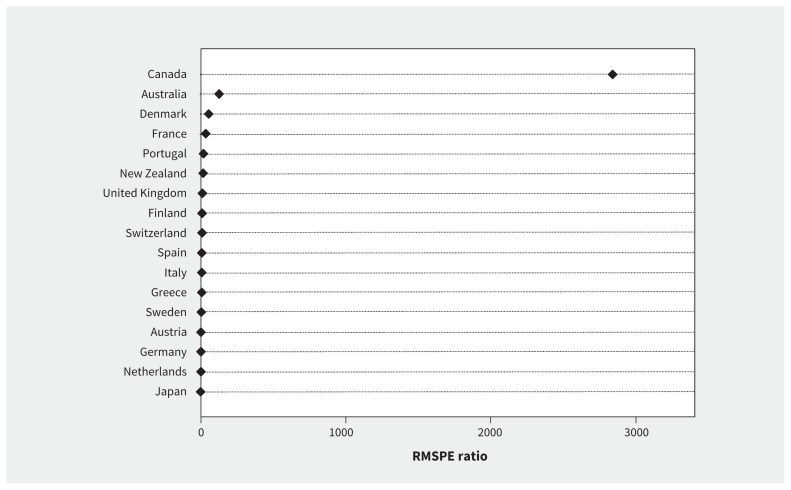
Ratio of post–North American Free Trade Agreement (NAFTA) root mean squared prediction error (RMSPE) to pre-NAFTA RMSPE: Canada and control countries. Note: RMSPE is the square root of the sum of the squared difference between the observed value in a country and its synthetic counterpart across a specified period. Here the RMSPE is estimated separately in the pre- and post-NAFTA periods, and the ratio shown above is the post-NAFTA RMSPE divided by the pre-NAFTA RMSPE.

Figure 2 and Appendix 10 (available at www.cmaj.ca/lookup/suppl/doi:10.1503/cmaj.161152/-/DC1) plot the results from the in-space placebo analysis of model robustness and show that the difference in supply before and after NAFTA far exceeds the difference in any other country.

We examined whether our choice of comparison countries could explain our results, by iteratively excluding each country from the sample, and by estimating our results including only OECD countries that were not European Economic Area members. The results were consistent with our main analysis, which suggests that our findings are not attributable to our choice of comparison countries and differences in trade instruments (Appendices 11–12, available at www.cmaj.ca/lookup/suppl/doi:10.1503/cmaj.161152/-/DC1). Further tests analyzed the potential influence on our results of predictor imbalance, serial correlation, a pre-intervention dip, changes in exports of other caloric sweeteners, changes to investment and unobserved factors that occurred when NAFTA was implemented (Appendix 12). The results did not qualitatively affect our results.

## Interpretation

We show that tariff reductions on HFCS-containing food and syrups in NAFTA were associated with a 41.6 (95% CI 25.06 to 58.21) kcal/capita daily increase in the supply in Canada of caloric sweeteners including HFCS. These findings were robust to additional sensitivity analyses, and are consistent with previous studies which find that countries enacting trade deals with the US experience changes in their food environments.^18^,^38^

Our study advances existing research in 3 important ways. First, we isolate a specific mechanism through which FTAs can affect diets: import tariffs. We have shown that a small and potentially inconspicuous change to tariffs can precipitate a substantial change to peoples’ diets, including increased consumption of HFCS. The population-wide consequences for public health are potentially enormous. This rise in HFCS consumption was correlated with a large rise in obesity rates, from 5.6% in 1985 to 14.8% in 1998.^39^ Rates of obesity among Canadians now rank among the highest of advanced industrialized nations that, unlike Canada, do not have trade agreements with the United States.^40^ The period after NAFTA also corresponded with rises in the prevalence of diabetes from 3.3% to 5.6%, from 1998/99 to 2008/09.^41^ Our findings are consistent with the hypothesis that US trade relations may have been an underlying population-level factor contributing to Canada’s comparatively high rates of obesity, diabetes and noncommunicable diseases, through increased population-level exposure to added sugars.

Second, our analysis provides more robust evidence that these associations are causal. We have used rigorous quasi-experimental methods to overcome potential confounding that is not addressed in the majority of previous research. Third, we identified that the rise in HFCS supply coincided with a decline in cane and beet sugar supply and a pause in the long-term decline in total sugar and sweetener supply. This suggests that total sugar and sweetener supply was higher in Canada after NAFTA than it might have been without NAFTA. This also suggests that trade agreements that apply greater tariff reductions on potentially hazardous food items may catalyze a “hazardous substitution effect,” in which populations replace less hazardous food items with more hazardous commodities that are subject to lower tariffs.

Our results have important implications for health policy. Trade agreements such as NAFTA are widely used macroeconomic policy instruments.^42^ NAFTA has been held up as a blueprint for future FTAs, including a potential new deal between the US and UK following its decision to leave the European Union, and the Transatlantic Trade and Investment Partnership between the US and the European Union, currently under negotiation.^43^,^44^ However, our analysis of the effects of NAFTA raises concern that new trade deals could harm population health should lower tariffs lead to increased supply and potential consumption of unhealthy food items, particularly those containing HFCS. FTAs may well yield benefits, via higher incomes and improved food security, but may also lead to numerous harms.^45^ Potential harms may be counteracted partially by targeted public health policies. Yet, our analysis shows that small and seemingly modest changes to tariffs can lead to a substantial rise in the supply of commodities like HFCS.

### Limitations

Our analysis has several limitations. First, our study analyzes changes in 1 specific product category in a developed country. This limits the generalizability of our findings, but enables us to parse the specific policies and mechanisms linking FTAs to changing diets. Second, data limitations preclude the possibility of fully testing how US exports responded to tariffs and the impact of changing HFCS consumption on health outcomes. These effects are debated, although there are concerns that the corn and soft-drink industries may distort evidence of the harms from HFCS.^46^ Fourth, supply may be an imperfect measure of consumption because of difficulties in estimating wastage and home production. Supply levels are nevertheless a widely used proxy for consumption and are especially appropriate in market equilibrium when supply and demand are equal.^47^–^50^ Fifth, causal inferences from our quasi-experimental study are limited, as with any statistical analysis with observational data. Finally, it is possible that 1 or more important events took place in Canada at the same time as NAFTA and accounted for our findings. We attempted to address this possibility using additional placebo studies and sensitivity analyses that did not qualitatively affect our results.

### Conclusion

These limitations notwithstanding, we find evidence that lower tariffs following NAFTA were associated with increased supply and likely consumption of caloric sweeteners in Canada including potentially hazardous HFCS. This has important implications elsewhere. This should be taken into account by countries negotiating future trade agreements.
